# Bouveret's syndrome: An old diagnosis. A modern multimodality approach (endoscopic and robotic surgical) of gastric outlet obstruction: Report of two cases

**DOI:** 10.1016/j.ijscr.2023.109134

**Published:** 2023-12-08

**Authors:** Tika Ram Bhandari, John Lin Hieng Wong, Jawad Ahmad, Khalid Akbari, Vinod Menon

**Affiliations:** a Department of Upper Gastrointestinal and Bariatric Surgery, University Hospitals Coventry and Warwickshire, Coventry, United Kingdom; b Department of Gastroenterology and Endoscopy, University Hospitals Coventry and Warwickshire, Coventry, United Kingdom; c Department of Hepatobiliary and Pancreatic Surgery, University Hospitals Coventry and Warwickshire, Coventry, United Kingdom

**Keywords:** Bouveret's syndrome, Gallstone disease, Endoscopy, Robotic surgery, Case report

## Abstract

**Introduction and importance:**

Bouveret's syndrome is an uncommon condition characterized by the impaction of a gallstone in the pylorus or duodenum via a cholecysto-enteric fistula causing gastric outlet obstruction. We report two unusual cases of Bouveret's syndrome causing gastric outlet obstruction in two elderly patients.

**Case presentation:**

Two elderly female patients presented to the surgical assessment unit with features of gastric outlet obstruction. In both cases, an urgent computed tomography (CT) of the abdomen showed pneumobilia, gastric distension, and gallstones impaction at the duodenal bulb. In Patient 1, endoscopic removal of the impacted gallstones was done successfully. She was discharged three days following an uneventful recovery. In Patient 2, an endoscopic removal of a single large gallstone was attempted, which was unsuccessful. She underwent robotic gastrotomy with extraction of the large gallstone with primary repair. She was discharged on 8th postoperative day.

**Clinical discussion:**

Treatment options for Bouveret's syndrome include endoscopic management and surgery. The selection of treatment options depends upon factors like the degree of obstruction, the impaction site, number, type or size of gallstones, patient co-morbidities and clinical parameters at presentation, as well as expertise available, both endoscopic and surgical.

**Conclusions:**

Bouveret's syndrome is one of the rare complications of gallstone. Endoscopic management can be effective at removing the impacted gallstones, which is particularly helpful for those elderly patients who have multiple medical co-morbidities, as in our first patient. Surgical management like minimal invasive surgery (robotic) can be beneficial in failed endoscopic attempt of removal of stone like in the second patient.

## Highlights

• Bouveret’s syndrome is one of the rare complications of gallstone disease.
• A modern multi-modalitity approach (endoscopic and robotic surgical) can be beneficial for even this rare disease.
• Awareness of this unusual entity is important for prompt management and better patient outcomes.

## 1 Introduction and importance

Bouveret's syndrome is a rare form of gallstone ileus characterized by the impaction of a gallstone in the pylorus or duodenum via a cholecysto-enteric fistula causing gastric outlet obstruction. Beaussier described Bouveret's syndrome (BS) for the first time in 1770. However, Leon August Bouveret was the first to report two cases in 1896 [1]. The prevalence of gallstone ileus represents 0.3–0.5 % of patients with gallstones, and Bouveret's syndrome occurs only in 1–3 % of gallstone ileus [2,3]. The morbidity and mortality rates of Bouveret's syndrome range from 12 to 30 % mainly due to the frailty of patients [4]. Effective management of Bouveret's syndrome needs a multi-modality approach based on the availability of treatment options as well as patient-specific factors. We report Bouveret's syndrome in two elderly female patients, managed with endoscopic stone removal and robotic surgery respectively, both with successful outcomes.

## 2 Method

We report this case in line with the updated consensus-based surgical case report (SCARE) guidelines [5].

## 3 Case presentation

### 3.1 Case 1

An 84-year-old female with a background history of diabetes mellitus and hypertension presented in the surgical assessment unit, with a 5-day history of nausea and multiple episodes of intermittent bilious vomiting. She reported intermittent episodes of upper abdominal pain for the last six months. She was diagnosed with acute cholecystitis secondary to gallstones three months ago. A computerized tomography (CT) scan of the abdomen and pelvis had shown contained gallbladder perforation, and at that time, she was managed conservatively. She underwent an endoscopic retrograde cholangiopancreatography (ERCP) one month ago, and a common bile duct stone was removed. She denied any previous abdominal surgery. On admission, she was ill-looking and dehydrated. Her vital signs were stable. Examination revealed a grossly distended abdomen, epigastric tenderness without peritoneal signs, and sluggish bowel sounds. On admission, blood tests showed raised white cell counts (WCC) 20 × 10^9^/L, c-reactive protein (CRP) 31 mg/L, alkaline phosphatase (ALP) 140 U/L, and normal alanine amino transferase (ALT) 12 U/L and total bilirubin 16μmol/L.

**Fig. 1:**
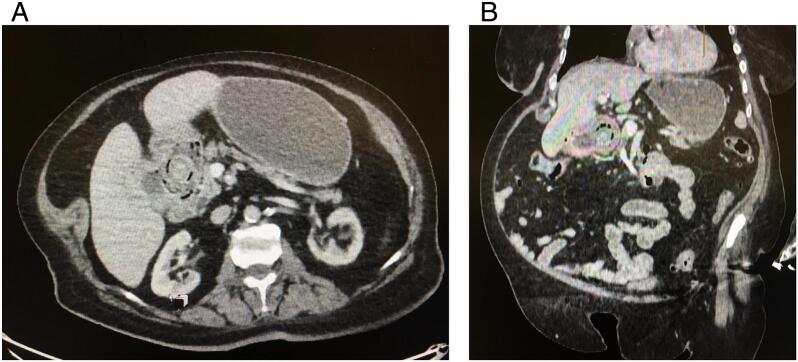
CT scan of the abdomen and pelvis showing artially-calcified gallstone(s) at the level of the superior duodenal flexure following cholecysto-duodenal fistulation causing upstream dilatation of the stomach suggestive of partial obstruction.

An urgent CT scan of the abdomen and pelvis showed a 2.8 cm partially-calcified gallstone (s) at the level of the superior duodenal flexure (Fig. 1A and B) following cholecysto-duodenal fistulation causing upstream dilatation of the stomach suggestive of partial obstruction, and some free air in the pericholecystic space. The latter was suggestive of an ongoing leak from either the biliary tree or duodenum, likely secondary to an imperfect seal around the fistulation. She was treated with intravenous fluid (normal saline), antibiotics, and nasogastric decompression (Ryles tube).

**Fig. 2:**
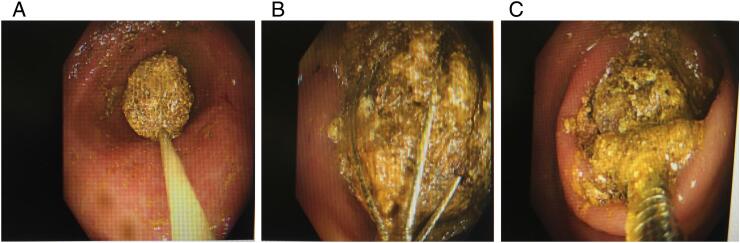
Endoscopy view showing several large, mixed soft and hard gallstones packing the duodenal bulb with the mechanical lithotripsy basket that was able to engage and crush the hard, or solid component of the impacted gallstones.

The patient underwent an urgent upper gastrointestinal (GI) endoscopy on the second day of admission. The procedure was performed in the endoscopy unit, with a gastroscope and conscious sedation. Initial findings revealed several large, mixed soft and hard gallstones packing the duodenal bulb (Fig. 2A, B, and C). The largest hard gallstone was estimated at 3 cm in diameter. A mechanical lithotripsy basket (LithoCrushV, Olympus) was used. The mechanical lithotripsy basket was able to engage and crush the hard, or solid component of the impacted gallstones. All the stone fragments were retrieved into the gastric lumen with the lithotripsy basket and Roth net (Boston Scientific), and removed successfully. At the end of the procedure, a cholecysto-duodenal (bulb) fistula was noted. Her blood reports gradually became normal post endoscopy. Her recovery was uneventful. She was discharged three days after admission with a follow-up plan in 6 weeks.

**Fig. 3:**
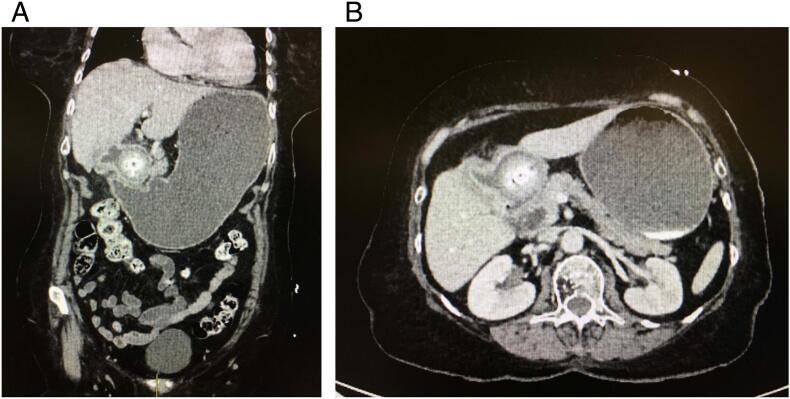
CT scan of abdomen and pelvis showing gastric outlet obstruction secondary to a large gallstone at the duodenal bulb.

**Table 1:** Laboratory parameters.

Parameters	On admission	Preop	Postop Day 1	Postop Day 3	Postop Day 5	Postop Day 7
WCC (10^9^/L)	7	5	8	7	6	6
CRP (mg/L)	72	6	77	278	152	86
ALP (U/L)	291	137	110	77	67	81
SGPT (U/L)	94	18	18	9	15	18
Total bilirubin (μmol/L)	4	5	5	3	3	3

Preop; preoperative, Postop; postoperative.

### 3.2 Case 2

An 80-year-old woman with a medical history of epilepsy admitted with the features consistent with bowel obstruction. She reported upper abdominal pain mainly in epigastrium for the last 2 weeks which was associated with nausea and multiple episodes of vomiting. The pain was aggravated with intake of food. She did not have any other significant medical and surgical history. Upon examination, she was dehydrated and tender upper abdomen noted. Upon admission an urgent CT scan of the abdomen and pelvis was done, that showed gastric outlet obstruction secondary to a large gallstone at the duodenal bulb (Fig. 3A and B). She was resuscitated with intravenous fluids, antiemetic and nasogastric decompression. Laboratory parameters were given in Table 1. An endoscopic attempt to remove the gallstone was made for the impacted stone. There was a large stone in duodenal bulb, unable to get past the stone to see lumen into second part of duodenum. Removal was tried with wire catheter. There was an ulcerated fistula tract visible partially from the bulb. The report indicated the stone was singular and too big. It was also not possible to place a naso-jejunal feeding tube to build up her nutrition. She was commenced on total parenteral nutrition (TPN). She developed a deep vein thrombosis in her right arm for which she was treated during admission with therapeutic heparin (enoxaparin). Three weeks after admission, she underwent surgery (Robotic assisted gastrotomy with extraction of calculus and primary closure) (Video). She was admitted to intensive care unit postoperatively for 5 days. She was treated with broad spectrum antibiotic. Once she was able to tolerate oral diet, TPN was stopped. She was discharged on 8th postoperative day when she opened bowels and was able to mobilize independently. She was clinically well when she was reviewed at 6-week follow up clinic appointment.

## 4 Clinical discussion

We report two uncommon cases of Bouveret's syndrome. Both patients were successfully treated - endoscopic crushing, retrieval of stones and removal of stone in the first patient and robotic gastrotomy in second patient. Bouveret's syndrome commonly presents with clinical features of gastric outlet obstruction. The presentation of Bouveret's syndrome can be non-specific. Clinical symptoms differ depending on the degree of obstruction, impaction site of the stone, and other various factors [6]. A history of gallstone, gallstone size >2.5 cm, female gender, and age of >60 years are common risk factors for Bouveret's syndrome [7]. Recurrent cholecystitis usually causes erosive necrosis of gallbladder wall that stimulates formation of cholecysto-duodenal fistula. Our first patient had a document history of cholecystitis due to gallstones which might have caused cholecysto-duodenal fistula.

The clinical diagnosis can be established by cross sectional imaging or direct endoscopic visualization of obstructing gallstones in the gastric outlet. Patients with gallstone ileus may show features of Rigler's triad (i.e., pneumobilia, small-bowel obstruction, and an ectopic gallstone) in radiological imaging [8]. A plain film of the abdomen may show these classic findings, up to only 21 % of cases. The sensitivity and specificity of contrast-enhanced CT for gallstone ileus are about 90–93 % and 100 % respectively [9]. Gastro-duodenal obstruction is an important finding in oesophago-gastro-duodenoscopy (OGD) which can be seen in all cases. Likewise, the obstructing stone and fistulous stoma are seen in 69 % and 13 % of cases respectively [6].

A uniform treatment protocol has not been postulated as it is a very rare condition. Multi-disciplinary team discussion and individualised modality of treatment is recommended. Various reports of successful endoscopic removal have been published in literature. A therapeutic gastroscopy (OGD) using endoscopic accessories (including mechanical lithotripsy baskets or Roth net) is comparatively straightforward to arrange, and effective at removing appropriately-sized gallstones. Laser lithotripsy, electrohydraulic lithotripsy, and extracorporeal shockwave lithotripsy are other methods of endoscopic approach. Common complications that can happen with attempted endoscopic stone removal are bleeding, perforation and gallstone ileus from stone migrating distally [8]. In our first patient, the hard gallstones impacted in the duodenum were crushed and retrieved successfully endoscopically without any complications.

A review of literature mentioned that 91 % of patients need surgery despite endoscopic treatment [10]. Surgery is the main form of treatment in conditions like gallstone pressing the duodenal wall, gallstone impaction in the fistula, and gastrointestinal hemorrhage [11]. Various surgical methods have been included in the literature including duodenotomy (or gastrotomy) alone; duodenotomy (or gastrotomy) with cholecystectomy and fistula closure (one-stage procedure); and duodenotomy (or gastrotomy) with cholecystectomy and repair of the bilio-digestive fistula after 4–6 weeks (two-stage procedure) [12]. In high risk cases, there is no evidence to suggest that doing cholecystectomy and fistula repair in the same episode improves outcomes as opposed to delayed surgery. Often this cohort of patients are comorbid with a high risk of surgery and hence relieving any obstruction is the priority. This is a similar scenario to gallstone ileus where the obstruction is relieved first and then a cholecystectomy is planned for later if the patient wishes and their comorbidities permit.

In our second patient, gastrotomy and removal of impacted gallstone with primary repair was performed by robotic approach. As we are a robotic centre, we usually utilize robots in almost all our major surgical procedures and complex cases. The utility of robots in cases like these improves visualization and make the procedure more precise to minimise the risks from surgery and better patient outcomes. This can definitely be achieved laparoscopically, however, using robot helps us to perform this procedure at lower pneumperitoneal pressures (8 mm Hg) which is particularly beneficial to minimise cardiovascular compromise in this cohort of patients with significant comorbidities and acutely unwell.

The outcome of Bouveret's syndrome mainly depends on the timing of diagnosis and prompt treatment. The outcome in both patients was entirely satisfactory. In view of rarity and complexity of this disease, the treatment strategy benefits from a multi-modality approach. Based upon possible morbidity and mortality rates of endoscopic versus surgical methods, the choice of treatment should be individualised to take into account patient factors like- age, comorbidities and performance status.

## 5 Conclusions

Bouveret's syndrome is one of the rare complications of gallstone without a uniform protocol of management. Endoscopic management can be effective at removing the impacted stones, which is helpful particularly for those elderly patients with multiple medical co-morbidities. Surgical management like minimal invasive surgery (robotic) can be beneficial in failed gastroscopic trial of removal of stone like in our second patient.

The following is the supplementary data related to this article.

Video Video showing intraoprative robotic assisted gastrotomy with extraction of calculus and primary closure. Video

## Ethical approval

The study required patient consent, we have taken consent from our patient for publication. Regarding ethical issues, this is a case study, not a research article. Therefore, the case report is exempted from ethical approval in our institution.

## Funding

This research did not receive any specific grant from funding agencies in the public, commercial, or not-for-profit sectors.

## Author contribution

1. TRB - study concept or design, data collection, literature search, writing paper, final decision to publish
2. JLHW – Study concept or design, data collection, literature search, writing paper, final decision to publish
3. JA - Study concept or design, data Collection, Literature search, final decision to publish
4. KA - Study concept or design, Literature search, final decision to publish
5. VM - Study concept or design, Literature search, writing paper, final decision to publish

## Guarantor

Tika Ram Bhandari

## Research registration number

Not applicable.

## Provenance and peer review

Not commissioned, externally peer-reviewed.

## Consent for publication

Written informed consent was obtained from the patient for publication of this case report and accompanying images. A copy of the written consent is available for review by the Editor-in-Chief of this journal on request.

## Declaration of competing interest

All authors declare that they have no competing interests.
